# DNA hypomethylation during MSC chondrogenesis occurs predominantly at enhancer regions

**DOI:** 10.1038/s41598-020-58093-5

**Published:** 2020-01-24

**Authors:** Matt J. Barter, Catherine Bui, Kathleen Cheung, Julia Falk, Rodolfo Gómez, Andrew J. Skelton, Hannah R. Elliott, Louise N. Reynard, David A. Young

**Affiliations:** 1 Skeletal Research Group, Biosciences Institute, Newcastle University, Central Parkway, Newcastle upon Tyne, NE1 3BZ UK; 2 UMR 7365 CNRS-Université de Lorraine, MolCelTEG Team, Biopôle de l’Université de Lorraine-Campus Brabois Santé, Vandoeuvre-lès-Nancy, France; 3 Bioinformatics Support Unit, Newcastle University, Newcastle upon Tyne, NE2 4HH UK; 4 Musculoskeletal Pathology Group, Institute IDIS, Servicio Galego de Saúde, Santiago de Compostela, Spain; 5 MRC Integrative Epidemiology Unit at the University of Bristol, Bristol, BS8 2BN UK; 6 Population Health Sciences, Bristol Medical School, University of Bristol, Bristol, BS8 2BN UK

**Keywords:** Cartilage development, DNA methylation, Mesenchymal stem cells

## Abstract

Regulation of transcription occurs in a cell type specific manner orchestrated by epigenetic mechanisms including DNA methylation. Methylation changes may also play a key role in lineage specification during stem cell differentiation. To further our understanding of epigenetic regulation in chondrocytes we characterised the DNA methylation changes during chondrogenesis of mesenchymal stem cells (MSCs) by Infinium 450 K methylation array. Significant DNA hypomethylation was identified during chondrogenic differentiation including changes at many key cartilage gene loci. Integration with chondrogenesis gene expression data revealed an enrichment of significant CpGs in upregulated genes, while characterisation of significant CpG loci indicated their predominant localisation to enhancer regions. Comparison with methylation profiles of other tissues, including healthy and diseased adult cartilage, identified chondrocyte-specific regions of hypomethylation and the overlap with differentially methylated CpGs in osteoarthritis. Taken together we have associated DNA methylation levels with the chondrocyte phenotype. The consequences of which has potential to improve cartilage generation for tissue engineering purposes and also to provide context for observed methylation changes in cartilage diseases such as osteoarthritis.

## Introduction

Our skeleton acts as an essential framework for the overall structure of our body. Accurate generation of this frame is dependent upon carefully controlled chondrocyte differentiation and cartilage formation, processes that underpin the development of the long and short bones of the skeleton^1^. Chondrocytes, the sole cartilage cell type, develop from condensations of mesenchymal progenitor cells in a process known as chondrogenesis^2^. During chondrogenesis progenitors commit to a tightly coordinated chondrocyte differentiation program determined by temporal and spatial expression of multiple growth factors and transcription factors^3^.

Epigenetic mechanisms, such as DNA methylation and histone modifications, provide cell-type specific regulation of gene expression essential for differentiation and maintenance of cell phenotype^4^. DNA methylation is a reversible process catalysed by DNA methyltransferases (DNMTs) on the fifth carbon of cytosine residues at CpG dinucleotides to form 5-methylcytosine (5mC)^5^. DNA methylation at gene promoter or enhancer sequences is frequently associated with gene repression where it correlates with the presence of inhibitory histone modifications and prevents the binding of transcription factors^6^. Loss of DNA methylation occurs passively during cell replication or by conversion of 5mC back to cytosine via oxidised intermediates with the assistance of ten eleven translocation (TET) proteins^7^.

During development and cell differentiation DNA methylation is dynamic, correlating with changes in gene expression^8,9^. The role, whereabouts and dynamics of DNA methylation during chondrogenesis remains poorly understood. In chondrocytes the regulation of genes such as MMP13, IL1, iNOS, chondromodulin, collagen 9 and GDF5 is influenced by DNA methylation at specific CpGs^10–15^. Similarly DNA methylation may regulate gene expression during chondrogenesis where COL10A1 induction correlates with its promoter demethylation, and intereference with DNA methylation during chondrogenesis by treatment with 5-aza-C alters gene expression^16,17^. While addition of methylation by DNMT3A has been found to regulate SOX9 expression in limb bud mesenchymal cells, and by DNMT3B to regulate cartilage metabolism and homeostasis^18,19^.

At the genome-wide level histone modifications are regulated during chondrogenesis and found to correlate with gene expression^20^. In contrast DNA methylation at gene promoters, assessed by reduced representation bisulfite sequencing (RBBS), did not correlate with gene expression^20^. Utilising the Infinium 450 K methylation array the effect of ageing on cartilage DNA methylation and the similarity between MSC-derived cartilage methylation in comparison with cartilage engineered from articular chondrocytes have been studied^21,22^.

Herein we focus directly on the differentially methylated CpGs during chondrogenesis in order to better define the chondrocyte methylome. Methylation changes are contrasted with gene expression to infer causality, while chromatin state information is integrated in order to further characterise regions of dynamic methylation. Further, a chondrocyte-specific methylation profile is established by comparison with cartilage and non-cartilage tissue methylation profiles, and the alterations in DNA methylation in osteoarthritis correlated. Interpretation of the epigenetic changes during chondrogenesis can provide context for those seen during deterioration of chondrocyte function in diseases such as osteoarthritis and also improve understanding of the processes governing differentiation of MSCs into chondrocytes for tissue regeneration purposes.

## Results

**Figure 1:**
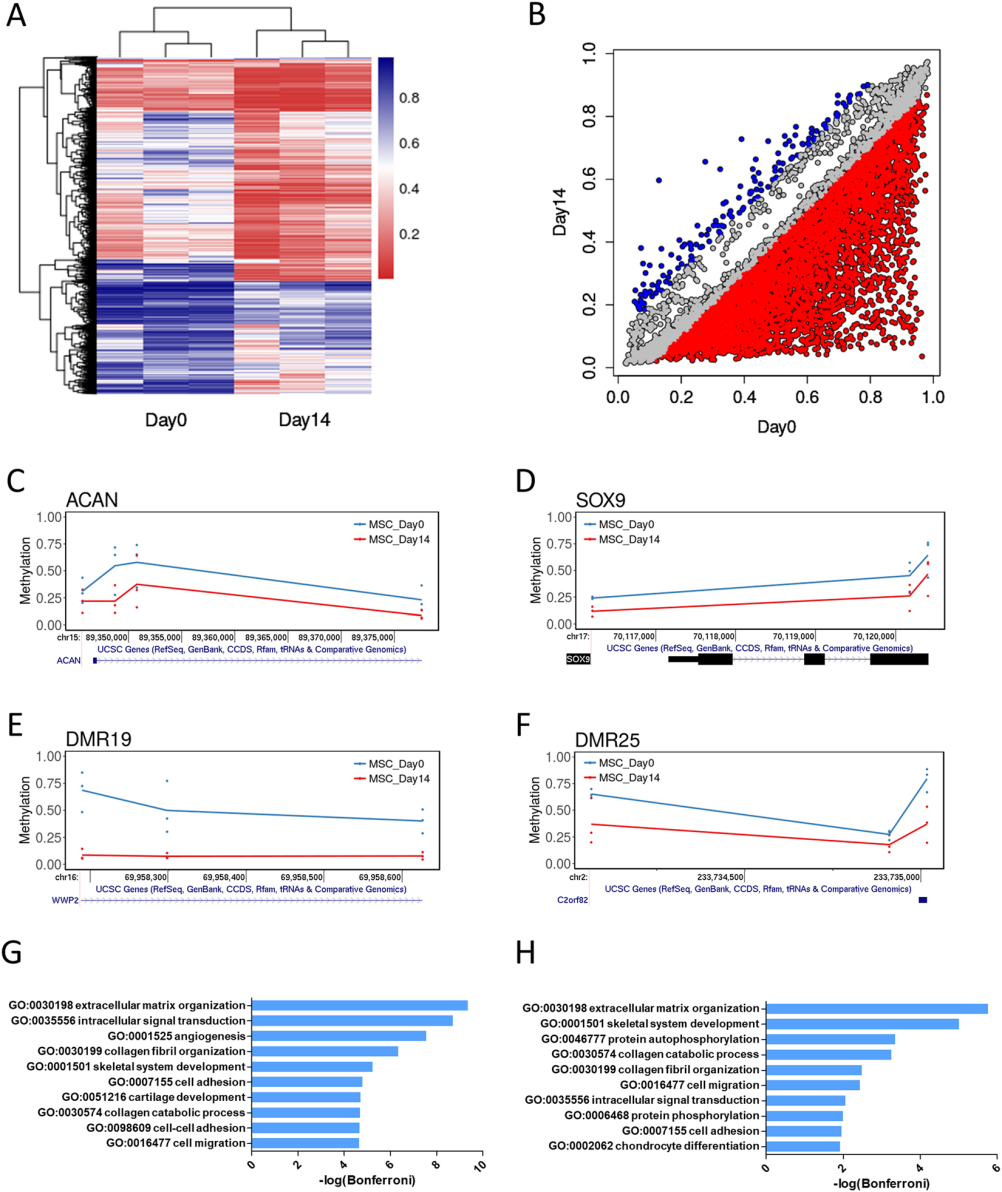
Differentially methylated CpGs during MSC chondrogenesis. (**A**) Heatmap of significant CpGs in MSC Day0 and Day14. (**B**) Correlation plot of significant CpGs Beta values at Day0 and Day14. Red indicates >10% hypomethylation, blue >10% hypermethylation at Day14. (**C**–**F**) Genomic vignette of significant CpGs at (**C**) ACAN and (**D**) SOX9 loci; or significant DMRs (**E**) DMR19 at WWP2 and (**F**) DMR25 at C2orf82 loci. Each donor is represented by a single dot and the average methylation for each timepoint represented by a line. Blue for MSC Day0, red for MSC Day14. (**G**,**H**) GO term analysis of (**G**) DML and (**H**) DMR genes.

Human MSCs were differentiated into chondrocytes by culture in chondrogenic differentiation medium for 14 days in a scaffold-free Transwell insert. A cartilage disc is formed with a homogenous extracellular matrix and concomitant rapid upregulation of chondrocyte gene expression indicative of differentiation, which is highly reproducible between different MSC donors^23,24^. In order to identify CpG methylation changes during MSC chondrogenic differentiation a DNA methylation profile was generated with the Infinium HumanMethylation450 BeadChip. There was no change in median methylation level of all CpG sites between MSCs (Day0) and MSC-derived chondrocytes (Day14). However, differential methylation analysis identified 5950 differentially methylated CpG loci (DMLs) with a significant, greater than 10%, change in methylation level at Day14 compared with Day0 (Fig. 1A and Supplementary Table 1). The vast majority of these (5802 DMLs) become hypomethylated during chondrogenesis, and the extent of methylation change is greater for CpGs becoming hypomethylated compared to hypermethylated (Fig. 1B). A number of DMLs are found at key cartilage gene loci such as ACAN and SOX9 (Fig. 1C,D). 12 of the DMLs associated with genes exhibiting significant expression changes during chondrogenesis were selected for pyrosequencing analysis in an independent chondrogenic differentiation of MSCs (Supplementary Fig. 1)^23^. Significant hypomethylation was confirmed for CpGs at all the associated genes, besides ACAN owing to the absence of hypomethylation in one MSC donor. Despite the variation in donor methylation the extent of methylation change per CpG during chondrogenesis are moderately correlated between the different MSC donors (Supplementary Fig. 2).

Grouping of DMLs in differentially methylated regions (DMRs) are considered an indication of regions with the potential to regulate gene transcription^25^. We defined DMRs as regions containing >1 differentially methylated CpG with a maximum separation of 1000 bp. 1276 DMRs are found during MSC chondrogenesis (Supplementary Table 2), including a DMR found at the promoter of SNORC (C2orf82), the transcript of which we found previously as the most upregulated during MSC chondrogenesis, and a DMR in WWP2 a key cartilage development protein also upregulated during chondrogenesis (Fig. 1E,F)^23,26^. GO term analysis of the genes with which these DMLs and DMRs are associated identifies terms consistent with the differentiation of cells into chondrocytes (Fig. 1G,H).

**Figure 2:**
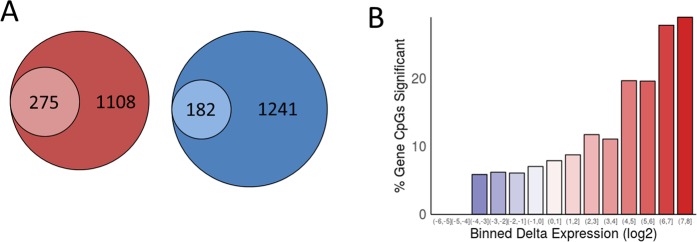
Comparison between methylation changes and gene expression changes. (**A**) Proportion of differentially expressed genes during MSC chondrogenesis with methylation changes. Outer circle represents total 2fold up- (red) or down- (blue) regulated gene number in chondrogenesis. Inner circle represents number of genes with at least 1 significant CpG. (**B**) Histogram showing enrichment of significant CpGs in comparison to direction and magnitude of binned log2 gene expression changes.

We previously identified expression changes in greater than 2000 genes during MSC chondrogenesis^23^. Intersection of DNA methylation changes with gene expression changes identifies DMLs at 25% of upregulated genes and 15% of downregulated genes (Fig. 2A). There is a greater enrichment of DMLs in the more upregulated genes (Fig. 2B).

**Figure 3:**
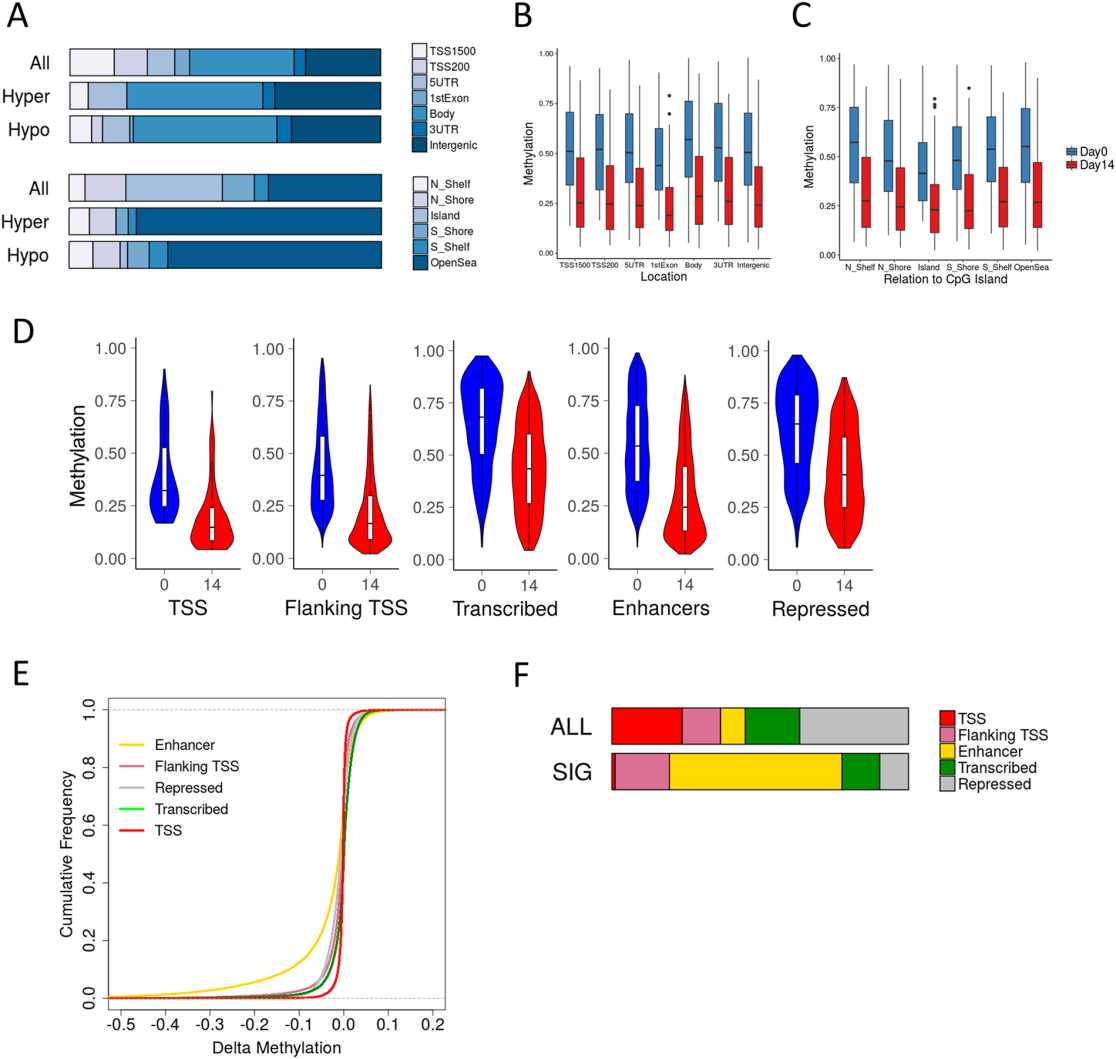
Genomic features and chromatin state of DMLs. (**A**) Proportion of significant hypo- and hyper-methylated CpGs overlapping gene and CpG island features. The distribution of all CpG probes on the array are presented for comparison. (**B**,**C**) Average methylation of significant hypo- and hyper-methylated CpGs overlapping (**B**) gene and (**C**) CpG island features in MSC Day0 and Day14. (**D**–**F**) Roadmap Epigenomics project MSC-derived chondrocyte-specific chromatin states were collapsed into five functional categories: TSS, Flanking TSS, Transcribed, Enhancers and Repressed. (**D**) Average methylation of DMLs overlapping the five chromatin state categories in MSC Day0 and Day14. (**E**) Cumulative frequency plot of methylation change at all CpGs during MSC chondrogenesis at each chromatin state category. (**F**) Proportion of DMLs overlapping chromatin state categories. The distribution of all CpG probes on the array are presented for comparison.

The context of the DMLs was further examined by examination of genomic features and chromatin states. In comparison with the distribution of probes across gene features in the array there is an enrichment of DMLs in gene body and intergenic regions (Fig. 3A). These DMLs are also less likely to be found in or around CpG island areas, where CpG islands are >200 bp in length with an observed/expected CpG ratio >60%. The hypomethylation occurs at all genomic features and locations, with shore CpGs up to 2 kb from a CpG island, shelf CpGs 2–4 kb, and open sea CpGs elsewhere in the genome (Fig. 3B,C). A 15 chromatin state model of MSC-derived chondrocytes has previously been generated from the integration of multiple histone mark ChIP-seq data as part of the Roadmap Epigenomics project^20^. CpG methylation levels during chondrogenesis were intersected with five functional categories of chromatin states from MSC-derived chondrocytes. Hypomethylation at DMLs was found at all chromatin states but was most extensive (>25%) for the enhancer state (Fig. 3D). Taking all CpGs into account an empirical cumulative distribution frequency plot showed that the enhancer state exhibited the greatest hypomethylation during chondrogenesis (Fig. 3E). Assessment of the distribution of DMLs across chromatin state categories indicates that a large proportion are found at enhancer states (Fig. 3F).

**Figure 4:**
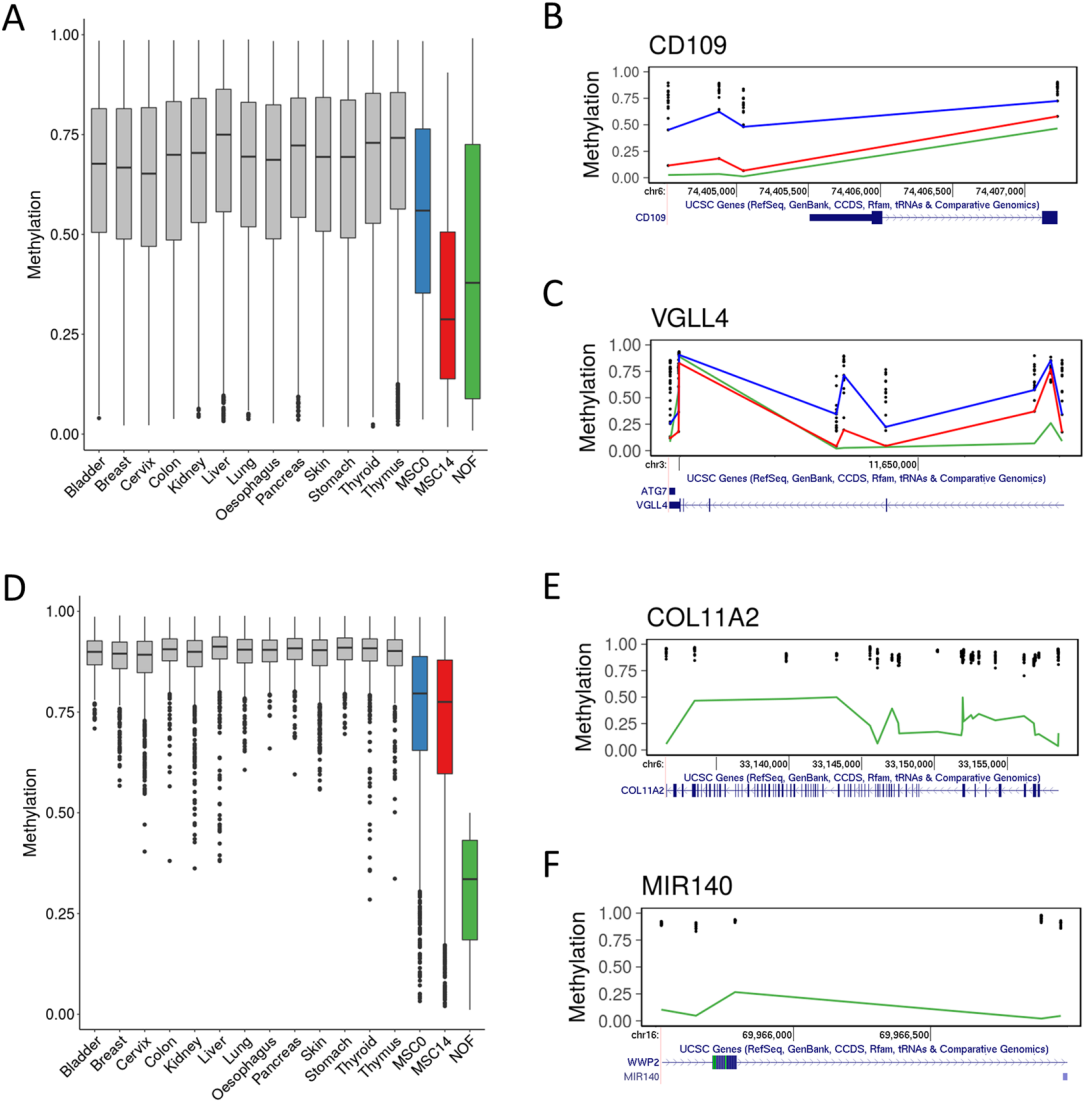
Methylation in chondrogenesis compared with human articular chondrocytes and other tissues. Mean methylation levels at Day0 and Day14, in human articular chondrocytes from neck of femur fracture patients (NOF), and in normal samples from a number of TCGA tissues were quantile normalised. (**A**) Average methylation of significant CpGs during MSC chondrogenesis in comparison with NOF and other tissues. (**B**,**C**) Genomic vignette of significant CpGs at (**B**) CD109 and (**C**) VGLL4 loci. (**D**) Methylation level of ‘cartilage-specific’ hypomethylated CpGs in NOF compared with other tissues. (**E**,**F**) Genomic vignette of ‘cartilage-specific’ hypomethylated CpGs at (**E**) COL11A2 and (**F**) MIR140 loci. (**B**,**C**,**E**,**F**) Each tissue is represented by a single dot. Lines indicate the methylation in MSC Day0 (blue), MSC Day14 (red), and NOF (green).

We previously identified DNA methylation levels in normal human hip chondrocytes from neck of femur fracture patients (GSE63695)^27^. The final methylation level of DMLs during MSC chondrogenesis were compared with CpG methylation levels in healthy cartilage (NOF) and a number of other tissues with methylation data available from TCGA (https://cancergenome.nih.gov/). The level of methylation at DMLs at Day14 is more similar to the level of methylation in human articular chondrocytes (NOF) than Day0 (Fig. 4A). In other tissues these DMLs have a higher level of methylation indicating a chondrocyte specific demethylation during chondrogenesis. For example DMLs at the CD109 and VGLL4 gene loci are methylated at levels similar to the other non-cartilage tissues at Day 0 but following chondrogenesis the methylation level is reduced to those found in articular chondrocytes (Fig. 4B,C). To further characterise the chondrocyte DNA methylome tissue-specific methylation markers were sought. 1464 CpGs were identified with an average methylation beta value < 0.5 in chondrocytes and >0.8 in >90% of tissues (Fig. 4D). These CpGs are more highly methylated in MSCs than cartilage, both before and after chondrogenesis. The COL11A2 and microRNA miR-140 gene loci include a number of CpGs exhibiting hypomethylation only in chondrocytes (Fig. 4E,F).

**Figure 5:**
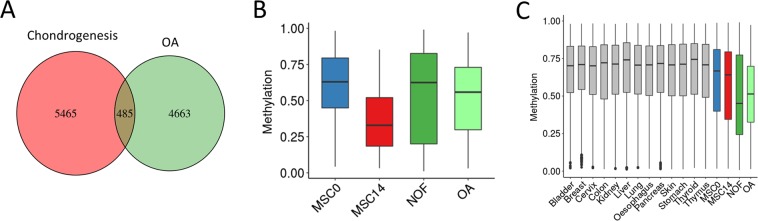
Overlap between DMLs in chondrogenesis and OA. (**A**) Overlap between differentially methylated CpGs in MSC chondrogenesis and in hip OA. (**B**) Average methylation of CpGs common to chondrogenesis and OA. (**C**) Average methylation of all significantly differentially methylated CpGs in OA compared with other tissues.

Changes in cartilage during OA may reflect a reinitiation of developmental pathways in the chondrocyte or a loss of chondrocyte phenotype due to disrupted epigenetic regulation^28,29^. Previously we identified DMLs between the hip cartilage of OA patients and healthy controls^27^. A comparison between CpGs altered in OA hip cartilage and MSC chondrogenesis indicated a small, but greater than expected, proportion (~10%, hypergeometric distribution p < 0.001) common to both (Fig. 5A and Supplementary Table 3). For these 481 common CpG there is a reduction in methylation during OA consistent with the changes during chondrogenesis (Fig. 5B). This is in contrast to the increase in mean methylation of all OA DMLs (Fig. 5C). In comparison with the methylation of other tissues many CpGs altered in OA are found at a lower level of methylation in chondrocytes (Fig. 5C).

## Discussion

DNA methylation establishes and reinforces cell-specific gene expression patterns during development and cell differentiation^9^. Here we show that DNA methylation changes, predominantly in the form of hypomethylation, are also a characteristic of differentiation of MSCs into chondrocytes during cartilage formation. These DMLs are enriched at enhancer regions consistent with the key role of cell-type specific enhancers in regulating gene expression. During MSC chondrogenesis the methylation level at significant CpGs becomes closer to the level found in articular chondrocytes, however significant differences remain between MSC-derived chondrocytes and adult chondrocytes.

By RBBS only limited changes in promoter methylation were identified during MSC chondrogenesis in a 3D alginate model^20^, but more recently by Infinium 450 K methylation array 1116 differentially methylated CpGs were identified across a timecourse of chondrogenic differentiation in a pellet model, albeit starting at Day14 (to Day49) in contrast to our comparison of undifferentiated vs. differentiated cells (Day0 vs. Day14)^21^. The vast majority of differentially methylated CpGs during chondrogenesis herein become hypomethylated indicative of a loss of repression at these locations. Although the impact of a defined percentage change in methylation will differ significantly on a CpG-specific basis there are clear inverse correlations between the extent of methylation and the level of gene expression^30,31^. Epigenome editing has also confirmed the direct effect of DNA methylation at selected CpGs on gene expression^30,32^. Transcription factor binding can cause local demethylation and thus the activation of the chondrocyte gene expression program might actively cause the loss of methylation^33^. Generally global levels of DNA methylation increase during differentiation, though this can vary in a lineage-specific manner^31,34,35^. At the gene level the shutdown of pluripotency genes occurs with increased methylation but the expression of lineage-specific genes is associated with hypomethylation^31,34,35^.

Interpretation of the genomic landscape and chromatin states harbouring significant CpGs indicated their enrichment in non-CpG rich enhancer regions of the genome. Consistent with the open-sea localisation of significant CpGs enhancers are mostly CpG-poor with variable methylation while in contrast 75% of promoters are within CpG islands with low methylation^33^. Hundreds of thousands of enhancers exist in the human genome, potentially contributing the major function of non-coding DNA regions^36^. These cis-regulatory regions can drive transcription over long distance from their target gene, by for example forming chromatin loops, and offer both cell- and developmental-stage type specific regulation of gene expression^37^. Cooperative action of transcription factors at enhancers and promoters facilitates chromatin access and activates gene expression^37^. DNA methylation and DMRs at enhancers have been shown to correspond to enhancer activity of differentiation and cell-type specific genes^8^. Numerous enhancers play key roles in regulating chondrocyte specific gene expression and skeletal development^38^. In particular both the regulation of SOX9 expression and the regulation by SOX9 are mediated by well-established enhancers^39,40^. During chondrogenesis the regulation of enhancer-associated H3K4me1 or H3K27ac modifications at distal cis-regulatory elements, up to 50 kb from transcription start sites, correlates with gene expression suggesting that enhancer regions regulate many upregulated genes^20^.

DMLs are enriched in genes associated with cartilage development GO terms, and grouping genes by the extent of their expression change during chondrogenesis indicated that the most upregulated genes contain more DMLs. However, many of the genes undergoing expression changes have no associated DNA methylation changes, and downregulated genes were also subject to hypomethylation. Consistent with this, hydroxymethylated cytosine (5hmC), a pathway intermediate generated during active demethylation, is found enriched at regulatory regions of genes during ATDC5 cell chondrogenic differentiation, although only a subset corresponded with changes in gene expression^41^. A number of factors may contribute to this disparity including the baseline methylation level and chromatin state, the particular transcription factors regulating expression, as well as the genomic location of CpGs on array. Interestingly the converse is also true whereby binding of transcription factors to promoters even in the absence of active transcription can cause the local loss of DNA methylation^33^. To better establish correlation whole genome methylation in combination with chondrocyte ATAC-seq data and gene expression would highlight the possible overlap of methylation and transcription factor binding sites^42^.

The disparity between MSC-derived cartilage and endogenous cartilage are well established owing to differences in lineage and developmental environment of the cells^43^. At the epigenetic level the DNA methylation profiles are also quite distinct^21^, however at many DMLs in chondrogenesis we identify a reduction in methylation level to the level found in adult articular chondrocytes, validating the utility of the MSC chondrogenesis model for chondrocyte differentiation and supporting the association of these loci with the chondrocyte phenotype. Notably these CpGs are found highly methylated in other tissues supporting the chondrogenesis-specific nature of the methylation changes. In particular the region containing WWP2 and miR-140 undergoes extensive demethylation during chondrogenesis and is found highly methylated in all other tissues further cementing the cartilage-specific function of these genes^26,44^. Genes with unexplored but possible functions in cartilage were also found hypomethylated in cartilage including CD109, a TGF-β co-receptor, and VGLL4 a regulator of Wnt signalling^45,46^. Previously methylation differences were also identified between MSC-derived cartilage from young and old donors however it is unclear whether these differences are due to baseline differences in the MSCs or as a result of differences in the differentiation capacity of the donors^22^.

Providing context for the changes in OA we found a small overlap between methylation changes in MSC chondrogenesis and those CpGs altered in OA, with these DMLs again enriched in enhancers^27^. Our data indicates that where there was previously no evidence of methylation changes in gene promoters during OA, such as ACAN and p21WAF1/CIP1, that dynamic methylation at enhancers regulating such genes should be considered^47,48^. A number of studies have also identified DNA methylation changes in OA or damaged cartilage but it remains to be determined whether these correspond to enhancer regions^49–55^. The subset of chondrogenesis DMLs also significantly altered in OA become hypomethylated consistent with the cells reactivating developmental pathways^28,29^. However the majority of CpGs in OA become hypermethylated and more similar to the level in other cell types indicative of a loss of chondrocyte identity. Synthesis of all OA DNA methylation studies by meta-analysis could provide a comprehensive catalogue of DMLs altered in disease for future comparison.

Despite the modest number of MSC donors used in this study our array analysis and pyrosequencing validation identifies consistent hypomethylation in the donors, although the extent of demethylation can vary significantly. Both male and female donors were used in this study, but sex chromosome CpGs are filtered from the analysis and we have not observed any sex specific differences in chondrocyte methylation previously^27^. We endeavoured to use MSC at low passage number and young donor age both for optimal chondrogenic differentiation and to limit the impact of epigenetic drift on the MSCs^56^. Many tissues and cell types experience an increase in methylation with age at a subset of CpGs^30,57^. Accordingly, the impact of age disparity between young MSC donors and aged NOF adult cartilage donors should be considered.

In conclusion, considerable demethylation changes to the epigenetic landscape occur during MSC chondrogenesis especially at sites marked by enhancer modifications. Comparison with other tissues, including healthy and OA cartilage, associates CpGs to the chondrocyte phenotype and provides context for changes in disease.

## Experimental Procedures

### Human bone marrow stem cell culture

Human bone marrow mesenchymal stem cells (MSC) (from four donors, 18–25 years of age) were isolated from human bone marrow mononuclear cells (Lonza Biosciences, Berkshire, UK) by adherence over 24 hours to tissue culture plastic and were expanded in monolayer culture in Mesenchymal Stem Cell Growth Medium (Lonza) supplemented with 5 ng/ml fibroblast growth factor-2 (R&D Systems, Abingdon, UK). Cultures were maintained in a humid atmosphere of 5% CO2/95% air at 37 °C. Once cells reached confluence, they were passaged (P1) using Trypsin/EDTA at a split ratio of 1:3. Experiments were performed using cells between P2-P7. The phenotypes of all MSC donors were tested previously^23^.

### Chondrogenic differentiation

MSC were resuspended in chondrogenic culture medium consisting of high glucose DMEM containing 100 µg/ml sodium pyruvate (Lonza), 10 ng/ml TGF-β3 (PeproTech, London, UK), 100 nM dexamethasone, 1x ITS-1 premix, 40 µg/ml proline, and 25 µg/ml ascorbate-2-phosphate (all from Sigma-Aldrich, Poole, UK). 5 × 10^5^ MSC in 100 µl medium were pipetted onto 6.5 mm diameter, 0.4-µm pore size polycarbonate Transwell filters (Merck Millipore, Watford, UK), centrifuged in a 24-well plate (200 g, 5 minutes), then 0.5 ml of chondrogenic culture medium added to the lower well as described^24^. Media were replaced every 2 or 3 days up to 14 days. Donor 1 chondrogenesis was performed in triplicate yielding an R^2^ > 0.997 between the replicates. Consequently, Donor 2 and Donor 4 were measured in singlicate. Donor 3 provided an additional baseline Day0 sample. Further sample information is provided in Supplementary Table 4.

### DNA isolation, bisulphite treatment and methylation array

Genomic DNA was extracted from cells prior to the induction of chondrogenesis (Day0) and from Day14 cartilagenous discs by disruption in Invitrogen PureLink Genomic Digestion Buffer (Life Technologies, Paisley, UK) using a small disposable plastic pestle and an aliquot of Molecular Grinding Resin (G-Biosciences, St. Louis, MO) followed by proteinase K digestion for 1 hour at 37 °C then nucleic acid purification with Invitrogen PureLink Genomic DNA Kit according to manufacturer’s instructions (Life Technologies). 1 µg of genomic DNA was bisulphite converted using the EpiTect Bisulfite Kit (Qiagen, Manchester, UK). DNA methylation profiling of the samples was carried out by Cambridge Genomic Services (Cambridge, UK), using the Illumina Infinium HumanMethylation450 Beadchip array (Illumina Inc., San Diego, USA).

### Analysis of infinium 450 K methylation data

Methylation analysis was performed using the R/Bioconductor package Minfi. Raw IDAT files were read and preprocessed and probes were filtered for high detection p-value (P > 0.05), location on sex chromosomes and potential SNP contamination. Array normalization was carried out using the preprocessFunnorm function to generate M values for statistical testing and Beta values between 0 and 1, with 0 being unmethylated and 1 fully methylated, indicative of the percentage CpG methylation per probe. Differentially methylated probes were identified by fitting a paired linear model followed by statistical analysis using an empirical Bayes method (R/Bioconductor package Limma) then filtered by significance threshold (P < 0.05, F-test, after correction for multiple testing using the Benjamini–Hochberg method). Annotation of probes was performed with R/Bioconductor package Illumina HumanMethylation450kdb, with mapping to the hg19 genome build. Enriched gene ontology (GO) pathways were identified by interrogation of the annotated genes with significant CpGs with DAVID^58^. Differentially methylated regions (DMRs) were identified by R/Bioconductor package DMRcate. DMRs are defined as regions containing >1 differentially methylated CpG with a maximum separation of 1000 bp.

### Integration with gene expression

Gene expression was profiled by Illumina whole‐genome expression array for Day0 and Day14 MSC chondrogenesis triplicate biological samples and expression analysis was performed in the R/Bioconductor Limma package^23^. For genes with multiple probes gene expression values were averaged then intersected with DNA methylation Beta values associated with gene loci. Correlation of significant CpGs with gene expression was performed on binned log2 chondrogenesis gene expression changes to identify the percentage CpGs at gene loci with methylation changes.

### Chromatin states

To assign MSC-derived chondrocyte chromatin states to probes, we downloaded the NIH Roadmap Epigenomics Mapping Consortium E049 15-state chromHMM model and mapped probes based on their locations in the genome^59^ (http://www.roadmapepigenomics.org/). Roadmap chromatin states were collapsed into five functional states: Transcription Start Site (TssA, TssBiv), Flanking Transcription Start Site (TssAFlnk, BivFlnk), Transcribed (Tx, TxFlnk, TxWk), Enhancer (Enh, EnhG, EnhBiv), and Repressed (ZNF/Rpts, Het, ReprPC, ReprPCWk, Quies). All plots were generated using the ggplot2 package in R.

### Comparison with articular chondrocyte and TCGA normal tissue 450 K methylation data

Publicly available 450 K DNA methylation profiles were downloaded for human articular cartilage neck of femur fracture control (NOF) and osteoarthritic diseased (OA) (GEO GSE63695) and from The Cancer Genome Atlas (TCGA) for non-cartilage tissue healthy control samples (https://cancergenome.nih.gov/)^27^. All tissue (cartilage, TCGA and Day0/14 MSC chondrogenesis) Beta values underwent quantile normalisation. Chondrocyte-specific hypomethylated CpGs were defined as CpG sites with an average methylation beta value < 0.5 in NOF cartilage and >0.8 in >90% of TCGA non-cartilage tissues.

### Pyrosequencing

DNA was isolated from Day0 MSCs and Day14 cartilagenous discs as above then 500 ng bisulphite converted with the EZ DNA Methylation Kit (Zymo Research, California, USA) following the manufacturer’s instructions. Pyrosequencing PCR was performed using the PyroMark PCR Kit (Qiagen) in a 20 µl reaction volume in duplicate with 1 µl bisulphite-converted DNA following the manufacturer instructions. Pyrosequencing assays were designed using PyroMark assay design software 2.0 (Qiagen), and the sequencing was performed using a PyroMark Q24 Advanced platform (Qiagen) with the recommended kit, following the instructions of the manufacturer and as previously detailed^60^. Sequences of forward and biotinylated-reverse PCR primers and sequencing primers are listed in Supplementary Table 5.

## Supplementary information

Supplementary Information.

Supplementary Table 1.

Supplementary Table 2.

Supplementary Table 3.

## Data Availability

MSC chondrogenesis 450 K methylation data is deposited at GEO (GSE129266).
