# Supplementary material for: DNA hypomethylation during MSC chondrogenesis occurs predominantly at enhancer regions
Source: Sci Rep. 2020 Jan 24;10:1169. doi: 10.1038/s41598-020-58093-5 (PMC6981252; doi:10.1038/s41598-020-58093-5)
Supplement: Supplementary file 1 — Supplementary Information. [file 41598_2020_58093_MOESM1_ESM.pdf]

DNA hypomethylation during MSC chondrogenesis occurs predominantly at enhancer regions

Matt J. Barter[1], Catherine Bui[2], Kathleen Cheung[3], Julia Falk[1], Rodolfo Gómez[4], Andrew J. Skelton[3], Hannah R. Elliott[6], Louise N. Reynard[1], David A. Young[1]

Supplementary Figure 1. Validation of 12 DMLs by pyrosequencing

Methylation at Day0 and Day14 in four MSC donors measured by pyrosequencing for 12 DMLs. A. ACAN cg01706824. B. SOX9 cg19298400. C. C2ORF82 cg07623116. D. C2ORF82 cg13047596. E. COL11A2 cg27512176, cg12472351 and cg19628686. F. FMOD cg15824962. G. MIA cg25152942. H. EXT1 cg22271457. I. SERPINA3 cg08057786 and cg06190732. For some pyrosequencing assays additional adjacent CpGs were also measured (denoted by + or -). The sequence analysed is reproduced below the plot with the CpGs in bold and the original DML underlined. Significant differences between Day0 and Day14 were assessed by two-tailed paired Students *t*-test.

Supplementary Figure 2. Comparison of the extent of methylation changes per MSC donor during chondrogenesis

Beta value methylation change at Day14 for each MSC donor DML compared to the average beta methylation of all donors at Day0. A. Donor 1 vs Donor 2. B. Donor 1 vs Donor 3. C. Donor 2 vs Donor 3. Red indicates >10% hypomethylation, blue >10% hypermethylation at Day14 for both donors.

Supplementary Table 1. List of differentially methylated loci (DML) during MSC chondrogenesis

Column heading descriptions: 450K array CpG ID; Day0 average beta methylation; Day14 average beta methylation; change in methylation at Day14 vs Day0; adjusted P value; chromosome; base position; DNA strand; CpG island identifier; the relation to CpG island; the UCSC RefGene name; the UCSC RefGene accession number and the UCSC RefGene group in relation to gene loci.

Supplementary Table 2. List of differentially methylated regions (DMR) during MSC chondrogenesis

Column heading descriptions: DMRcate DMR number; DMR genomic location; chromosome; DMR start base position; DMR end base position; DMR size (bases); number of 450K array CpGs within DMR; minimum FDR of the smoothed estimate - where FDR smoothing results in increased power and cleaner spatial separation of signals from noise; Stouffer summary transform of the individual CpG FDRs – where the Stouffer method generates a more conservative combined statistic from independent significant test p values; maximum beta methylation change within the DMR; average beta methylation change within the DMR; gene promoters overlapping with the DMR.

Supplementary Table 3. Intersection of DML during MSC chondrogenesis and DML in osteoarthritis

Column heading descriptions: 450K array CpG ID; Day0 average beta methylation; Day14 average beta methylation; change in methylation at Day14 vs Day0; adjusted P value; chromosome; base position; DNA strand; CpG island identifier; the relation to CpG island; the UCSC RefGene name; the UCSC RefGene accession number and the UCSC RefGene group in relation to gene loci.

Supplementary Table 4. Sample information

Supplementary Table 5. Pyrosequencing primers

Sequences of forward, biotinylated-reverse (*) and sequencing primers for pyrosequencing including the sequence to analyse for the PyroMark Q24 Advanced platform.

Supplementary Figure 1

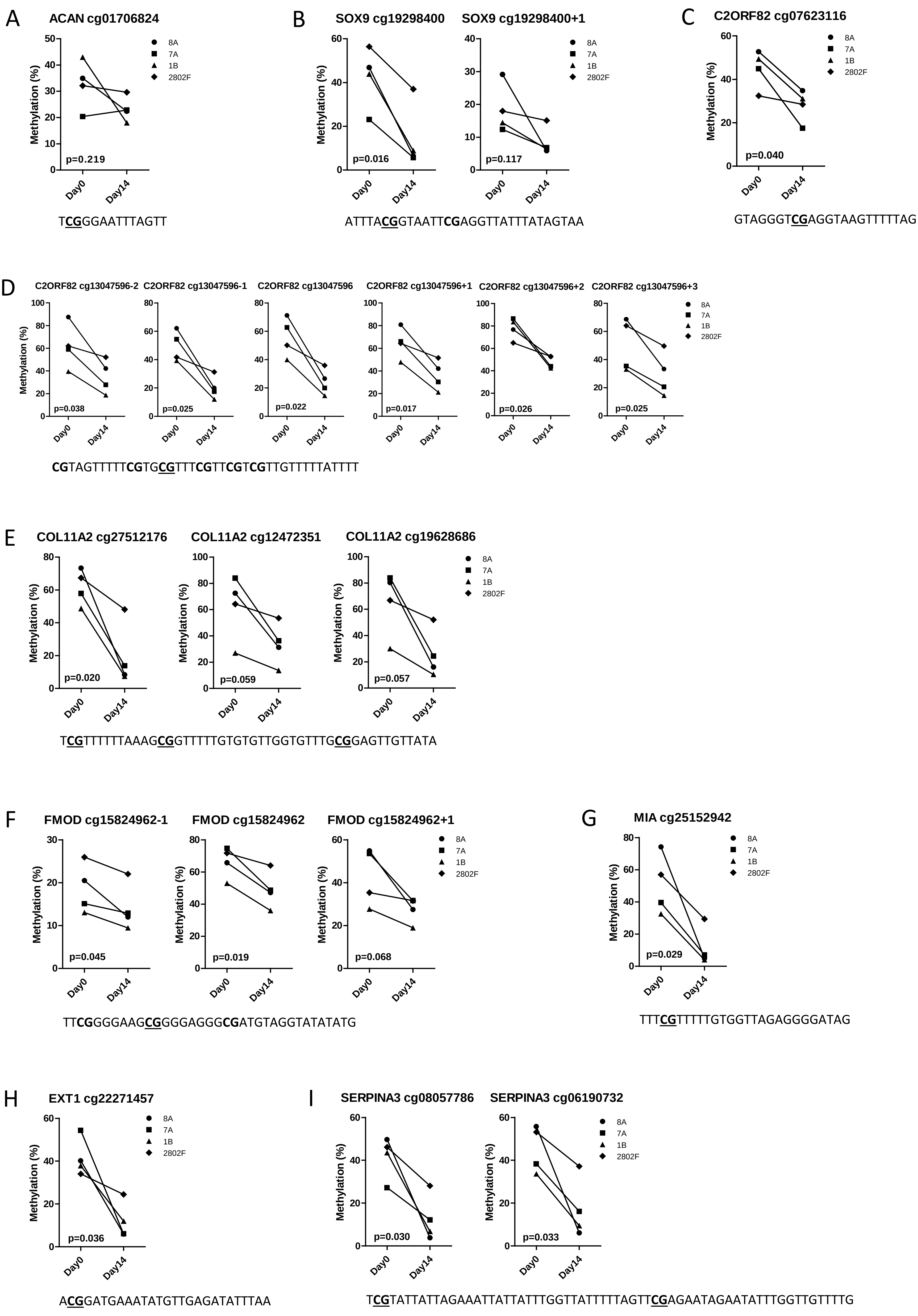

Supplementary Figure 2

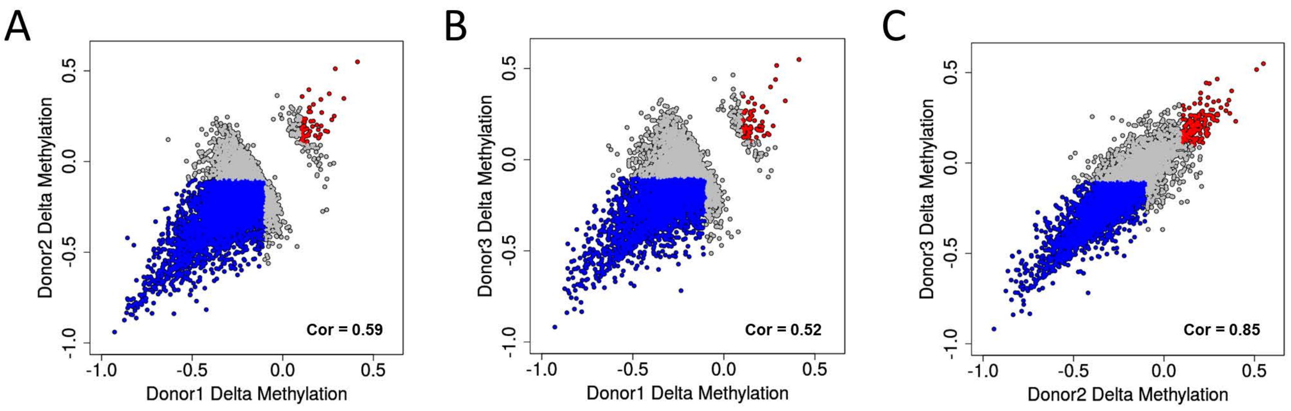

Supplementary Table 4

Samples	Sex	Age	Treatment
MSC_Donor1_Day0_1	F	22	Day0 undifferentiated
MSC_Donor1_Day0_2	F	22	Day0 undifferentiated
MSC_Donor1_Day0_3	F	22	Day0 undifferentiated
MSC_Donor1_Day14_1	F	22	Day14 chondrogenic
MSC_Donor1_Day14_2	F	22	Day14 chondrogenic
MSC_Donor1_Day14_3	F	22	Day14 chondrogenic
MSC_Donor2_Day0_1	F	24	Day0 undifferentiated
MSC_Donor1_Day0_4	F	22	Day0 undifferentiated
MSC_Donor3_Day0_4	F	24	Day0 undifferentiated
MSC_Donor4_Day14_1	M	22	Day14 chondrogenic
MSC_Donor2_Day0_2	F	24	Day0 undifferentiated
MSC_Donor2_Day14_1	F	24	Day14 chondrogenic

Supplementary Table 5

CpG	Primer	Primer sequence	
**ACAN cg01706824**	F	TTTTTTTGGGATACTGGAGTTAAATATATAA	
	R	*CTAACAACCCCCTCTCACT	
	Sequencing	GTGTTAGGGATGATAAATTTT	Sequence to analyze: TYGGGAATTT AGTT
**SOX9 cg19298400**	F	ATTAGATATTTTAGGGTTGGAATTTTAAGT	
	R	*CCCTCCTACCTCTACAAATAAATATCAT	
	Sequencing	AGGGTATTTTTGGTTTATATG	Sequence to analyze: ATTTAYGGTA ATTYGAGGTT ATTTATAGTA A
**C2ORF82 cg07623116**	F	GGGAGTTTAGTTAAGGTTAGTGGATTGAA	
	R	*TCCCACCTCTAAACCCTCAAAATTCTATCA	
	Sequencing	TGGGTGGGAAGTTGA	Sequence to analyze: GTAGGGTYGA GGTAAGTTTT TAG
**C2ORF82 cg13047596**	F	TGTTTTTAGGTTTAGGAAAGTAGGT	
	R	*CAAAACCAAACAAAATACCATCCTAAC	
	Sequencing	TTTTTATTGTTTTGAAGTTAATTAG	Sequence to analyze: YGTAGTTTTT YGTGYGTTTY GTTYGTYGTT GTTTTTATTT T
**COL11A2 cg27512176, cg12472351 and cg19628686**	F	TGTGGGTAGGTTGTTTATATGAT	
	R	*AACCCCCACCTAAACCTAAC	
	Sequencing	TTTGTGTTTTTGTTTTATTTATTTT	Sequence to analyze: TYGTTTTTTA AAGYGGTTTT TGTGTGTTGG TGTTTGYGGA GTTGTTATA
**FMOD cg15824962**	F	TGGAAATAAGTGTTTAGAAAGTTGTAAGT	
	R	*CTTCCTACCCTCTAATCTCTCT	
	Sequencing	AATTAGGTGGTGATTATATT	Sequence to analyze: T TYGGGGAAG YGGGGAGGGY GATGTAGGTA TATATG
**MIA cg25152942**	F	TGGGATTTTGTTTAGTTTAAGGTTTAGTA	
	R	*ATTCAATTCCATCTTCTCTTAAATTAACT	
	Sequencing	GGTGGTTTTTTTTATAGGTTT	Sequence to analyze: TTTYGTTTTT GTGGTTAGAG GGGATAG
**EXT1 cg22271457**	F	ATGTAGGGGATAGTTATTATTGTGTTTA	
	R	*TTCTAAATTTTCCTAAAAAATCCAAATTAT	
	Sequencing	GGTGAAATTTTTGAGATGAG	Sequence to analyze: AYGGATGAAA TATGTTGAGA TATTTAA
**SERPINA3 cg08057786 and cg06190732**	F	GTTTTTTTGATTTTGGAGGAAGAGA	
	R	*CAAAAAACTCCTCCCCCTACTT	
	Sequencing	ATTTGAGTAGGTTTAATAAGT	Sequence to analyze: TYGTATTATT AGAAATTATT ATTTGGTTAT TTTTAGTTYG AGAATAGAAT ATTTGGTTGT TTTG
